# Adropin and Irisin Deficiencies Are Associated With Presence of Diagonal Earlobe Crease in CAD Patients

**DOI:** 10.3389/fcvm.2021.719763

**Published:** 2021-10-12

**Authors:** Na Wei, Ronghuai Zhang, Zhanfang Zhu, Rui Li, Qi Yu, Qingyu Wang, Cuixiang Xu, Meijuan Ma, Shengzhi Mu, Fuqiang Liu, Junkui Wang

**Affiliations:** ^1^Department of Cardiovascular, Shaanxi Provincial People's Hospital, Xi'an, China; ^2^Xi'an Jiaotong University Hospital, Xi'an, China; ^3^Shaanxi Provincial Clinical Research Center for Geriatric Medicine, Shaanxi Provincial People's Hospital, Xi'an, China; ^4^Shaanxi Key Laboratory of Ischemic Cardiovascular Diseases, Institute of Basic and Translational Medicine, Xi'an Medical University, Xi'an, China; ^5^Shaanxi Provincial Key Laboratory of Infection and Immune Diseases, Shaanxi Provincial People's Hospital, Xi'an, China; ^6^Department of Burn and Plastic Surgery, Shaanxi Provincial People's Hospital, Xi'an, China

**Keywords:** diagonal earlobe crease, adropin, irisin, atherosclerosis, endothelial dysfunction

## Abstract

**Background and Aims:** Diagonal earlobe crease (ELC) has been considered a potential cutaneous marker of atherosclerosis. However, the potential mechanism by which ELC and atherosclerosis are linked has not been adequately defined. Roles of adropin and irisin, novel biomarkers of endothelial function, in ELC have not been well-studied. This study aimed to test whether individuals with ELC are deficient in adropin and irisin, a characteristic that would likely promote endothelial dysfunction and provide a plausible common pathological basis for atherosclerosis and ELC.

**Methods:** Patients diagnosed with coronary artery disease (CAD) with (*n* = 45) and without (*n* = 45) ELC were consecutively enrolled in the study. The ages of the patients enrolled ranged from 40–70 years. Other patients (*n* = 45) without ELC or CAD were recruited as the control group. All patients underwent coronary angiography. Serum adropin and irisin concentrations were assessed *via* enzyme-linked immunosorbent assay.

**Results:** Circulating levels of irisin in the ELC group were significantly lower than those in the non-ELC group, and were highest in the control group. Serum adropin levels of the ELC group were significantly lower than those of the non-ELC group (*P* < 0.001). Interestingly, although the serum adropin level of the control group was greater than that of the non-ELC group, the difference failed to achieve statistical significance. In subgroup analysis of CAD and ELC, both serum adropin and irisin levels of the CAD and ELC groups were lower than those of the control group (*P* < 0.001). Receiver-operating characteristic curve analysis revealed that adropin and irisin have similar prognostic power for CAD and ELC.

**Conclusions:** Low adropin and irisin were significantly associated with CAD and ELC. The deficiencies in adropin and irisin may be a common cause of both atherosclerosis and ELC, which explains why patients with ELC are prone to CAD.

## Introduction

Coronary artery disease (CAD) is a leading cause of morbidity and mortality worldwide, conferring significant individual health and societal consequences (1). The presence of simple visible signs associated with early stages of CAD has attracted attention. Numerous epidemiological studies have demonstrated that visible signs, including baldness, ear lobe crease (ELC), and xanthelasmata, either alone or in combination, are associated with increased CAD risk in the general population, independent of well-known cardiovascular risk factors (2). The diagonal ELC, namely Frank's sign, was initially identified by Frank in 1973 (3). Patients with ELC have a diagonal fold or wrinkle-like line that extends from the tragus, across the lobule, to the rear edge of the auricle area of the ear. Many epidemiological and meta-analyses have demonstrated that ELC is independently linked to CAD, and the sign has been recognized as a simple cutaneous marker of cardiovascular disease (CVD) pre-disposition (4–10). However, the association between CVD and ELC remains unclear because pathophysiologic mechanisms linking the disease to the marker have not yet been elucidated (11).

The newly discovered polypeptide hormones adropin and irisin broadly impact energy metabolism and vascular homeostasis in humans. Adropin is a 76-amino acid, pluripotent peptide hormone that is encoded by the recently discovered energy homeostasis-related gene (ENHO) (12, 13). Adropin upregulates endothelial nitric oxide (NO) synthase expression and increases levels of nitric oxide release *via* vascular endothelial growth factor receptor 2 (VEGFR2)-phosphatidylinositol 3-kinase-AKt and VEGFR2-extracellular signal regulated kinase pathways. Further, it maintains insulin sensitivity and protects against obesity-associated hepatosteatosis (13). The 112-amino acid peptide irisin was initially considered a thermogenic protein that expends energy by converting white adipose tissue to brown (14). Current research suggests that irisin improves endothelial functioning by modulating the HO-1/adiponectin axis and the AMPK-eNOS pathway (15, 16). Endothelial dysfunction contributes to the development and progression of CVD (17), and several clinical studies have demonstrated that low serum levels of adropin and irisin are associated with CAD (18, 19).

The link between adropin, irisin, and ELC remains unclear. We hypothesized that the two hormones might provide a common pathophysiologic basis for the association between ELC and CAD. Thus, we conducted a cross-sectional study to test whether a deficiency of adropin and irisin is associated with CAD in patients with ELC.

## Materials and Methods

### Subjects

Patients from Shaanxi Provincial People's Hospital with (*n* = 45) and without ELC (*n* = 45) who underwent coronary angiography and were subsequently diagnosed with CAD were consecutively enrolled in the study from May to October of 2016. Other patients (*n* = 45) without ELC and CAD whose coronary arteries were determined to be healthy *via* coronary angiography or coronary computed tomography angiography were recruited. All patients included in the study were aged <70 years because the correlation between ELC and CAD in patients in that age range is weak. Because the heavy earrings may develop creased earlobes, the women who have pierced ear were ruled out in our study. All study participants were ethnically Chinese Han and received a brief medical questionnaire prior to an examination. The exclusion criteria were as follows: pierced ear, acute coronary syndrome, severe chronic heart failure, valvular heart disease, arrhythmia, chronic kidney disease, adrenal insufficiency, thyroid dysfunction, malignancy, use of steroids, systemic immune-mediated diseases, and non-steroidal anti-inflammatory or other immunosuppressive drugs. This study was approved by the ethics committee of Shaanxi Provincial People's Hospital. All patients signed informed consent to participate in the study.

### Definition of CAD

Coronary angiography was performed using a femoral or radial artery approach by an experienced team of cardiologists, and data were recorded at a rate of 15 frames/s. The definition of significant CAD was a luminal diameter of the major coronary artery or one of its major branches possessing >50% stenosis. Control patients had <50% stenosis within each of the major epicardial vessels. The decision regarding whether coronary arteries had significant levels of luminal stenosis was made visually by two independent investigators.

### Definition of ELC

**Figure 1:**
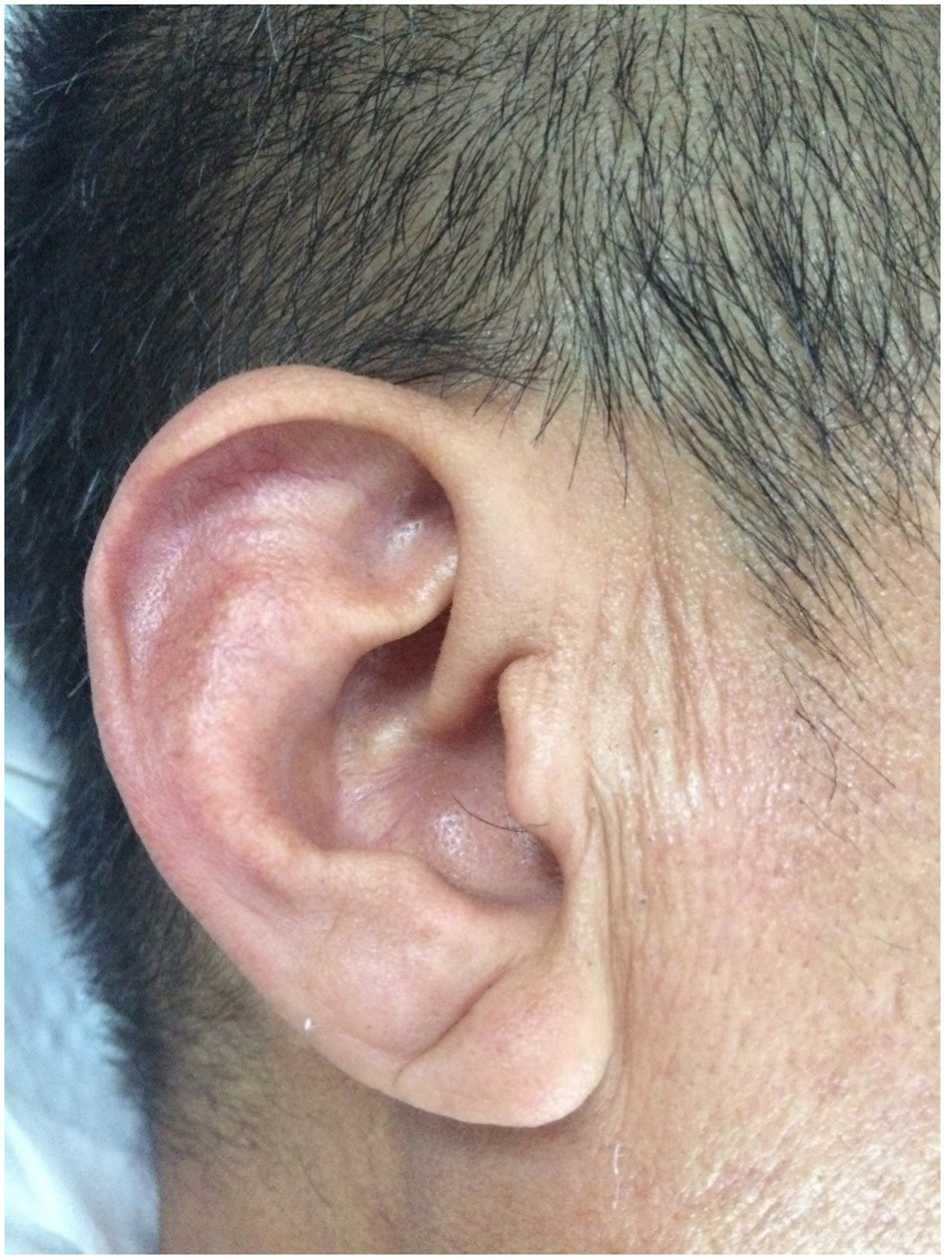
Typical example of a bilateral earlobe crease.

Previously published diagnostic criteria were adopted to determine the presence of an ELC (20). Briefly, the earlobe of each participant was first observed in the sitting position. Second, if there appeared to be an ELC, characteristics (e.g., length, depth, width, and number of creases) of each earlobe crease were recorded and evaluated. The length of the crease was required to span two-thirds or more of the earlobe. If the ELC appeared on at least one earlobe, we assigned the patient to the ELC group (Figure 1). Earlobes were digitally photographed for independent assessment by two researchers. Discrepancies were resolved by reaching a consensus *via* the participation of a third researcher.

### Biochemical Analyses

Fasting venous blood samples were withdrawn in EDTA-aprotinin tubes and immediately placed in an ice bath. Serum lipid levels such as total cholesterol, triglycerides, and HDL-C were measured *via* enzymatic procedures using an automated analyzer (AU 2700 Olympus, 1st Chemical Ltd., Japan). Commercial enzyme-linked immunosorbent assay kits were used to measure serum levels of irisin and adropin (Cusabio Biotech Co. Ltd., Wuhan, China). Intra- and inter-assay coefficients of variation were evaluated using five plasma samples of adropin or irisin.

### Statistical Analysis

Data were presented as means ± SD. Additionally, one-way analysis of variance was administered to determine differences between biochemical markers. Age, gender, and body mass index were adjusted *via* multivariable analysis. Fisher's exact-test and Wilcoxon rank-sum-test were performed using the stats package of R, version 3.6.3. Receiver-operating characteristic (ROC) curves were constructed with the ggplot2 and pROC package in R. The areas under the curves were calculated to obtain cutoff values for the diagnosis of CAD and ELC. Probability was assessed using a two-tailed *P*-value of <0.05 to indicate statistical significance.

## Results

### Profiles of Study Subjects

Table 1 includes the baseline characteristics of the study participants. The ages of the patients in each group ranged from 40 to 68 years (*P* > 0.05). No notable discrepancies were noted among groups regarding risk factors for atherosclerosis, such as hypertension, diabetes mellitus, dyslipidemia, obesity, and smoking status. Additionally, there was no difference in SYNTEX scores between the ELC group and non-ELC group (Supplementary Figure 1).

**Table 1:** Baseline demographic and clinical characteristics of the enrolled patients.

**Parameter**	**All patients (*n* = 135)**	**ELC group (*n* = 45)**	**Non-ELC group (*****N*** **=** **90)**		***p-*value**
			**CAD^**+**^ group (*n* = 45)**	**CAD^**−**^ group (*n* = 45)**	
Mean age (year)	58.3 ± 6.54	60.0 ± 6.69	57.38 ± 5.8	57.6 ± 6.86	0.109
Sex (m/f)	83/52	32/13	27/18	24/21	0.134
Body mass index, kg/m^2^	22.6 ± 3.0	23.0 ± 2.8	22.3 ± 2.9	22.5 ± 3.2	0.641
Systolic BP (mm Hg)	130.5 ± 17.1	130.7 ± 18.4	129.6 ± 17.4	131.2 ± 15.7	0.896
Diastolic BP (mm Hg)	80.9 ± 10.7	78 ± 9.34	81.8 ± 9.54	82.9 ± 12.5	0.077
HbA1c (%)	6.01 ± 0.75	5.88 ± 0.48	5.85 ± 0.53	6.32 ± 1.02	0.004
Urea nitrogen, mmol/L	5.0 ± 1.55	4.97 ± 1.59	4.74 ± 1.48	5.31 ± 1.54	0.213
Creatinine, mmol/L	72.6 ± 16.2	74.5 ± 19.7	72.0 ± 13.4	71.2 ± 15.0	0.620
Cystatin c, mmol/L	1.04 ± 0.04	1.06 ± 0.31	0.94 ± 0.25	1.11 ± 0.57	0.117
Total cholesterol, mmol/L	4.12 ± 1.18	4.14 ± 1.12	4.09 ± 1.45	4.12 ± 0.93	0.985
Triglycerides, mmol/L	1.57 ± 0.81	1.53 ± 0.83	1.58 ± 0.63	1.62 ± 0.94	0.882
LDL-cholesterol, mmol/L	2.16 ± 0.8	2.22 ± 0.87	2.13 ± 0.94	2.13 ± 0.55	0.821
HDL-cholesterol, mmol/L	1.06 ± 0.24	1.09 ± 0.29	1.04 ± 0.19	1.03 ± 0.22	0.449
Smoking status, (y/*n*)	80/55	30/15	26/19	24/21	0.564
Hypertension, *n* (%)	61 (45.2)	29 (64.4)	14 (31.1)	18 (40)	0.002
Diabetes mellitus, *n* (%)	37 (27.4)	9 (20)	14 (31.1)	14 (31.1)	0.11
SYNTEX score	–	9.64 ± 4.19	9.96 ± 2.67	–	0.676

### Circulating Levels of Adropin and Irisin in Patients With CAD

**Figure 2:**
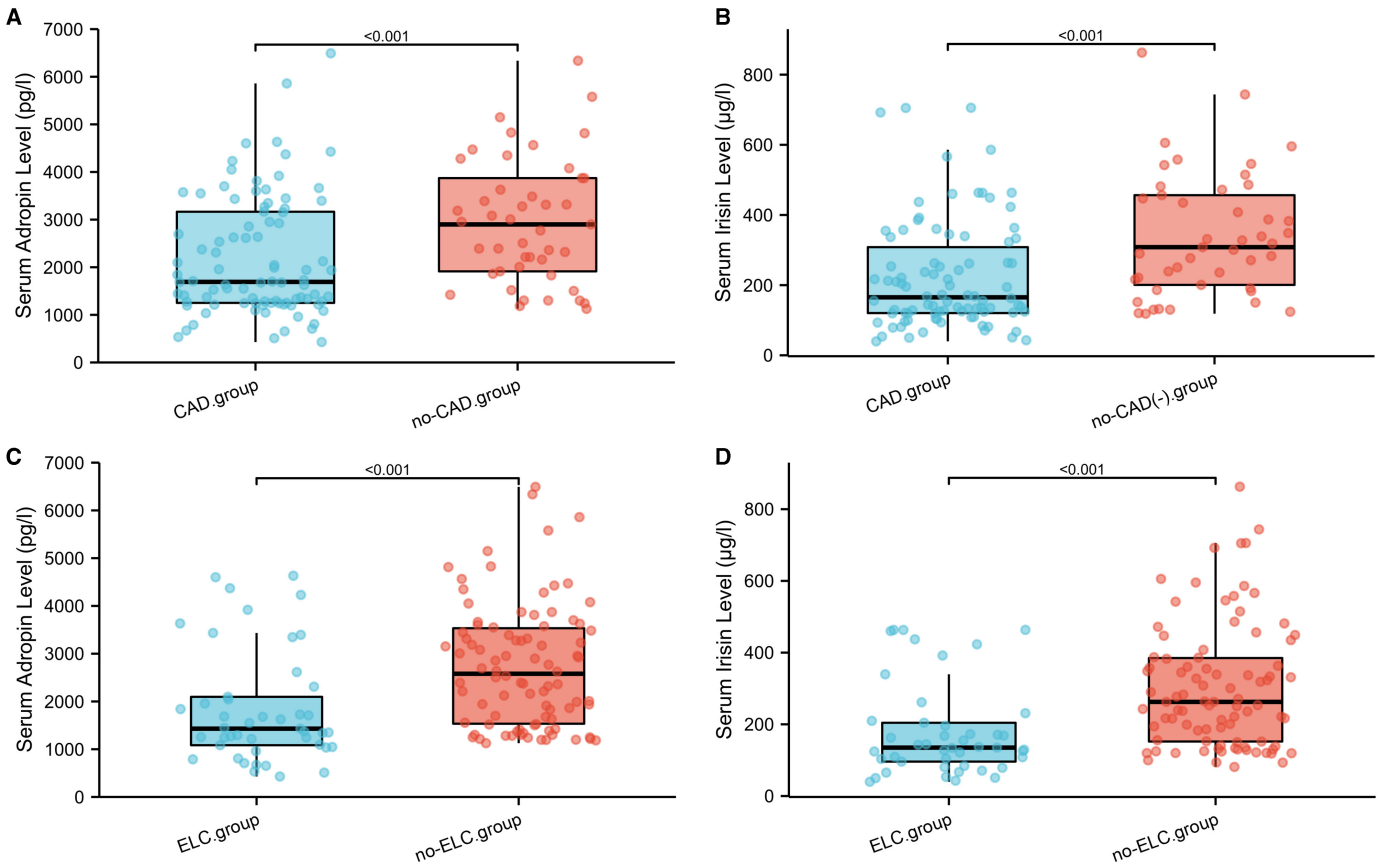
**(A–D)** Comparison of serum adropin and irisin levels in subgroup analysis on the basis of CAD and ELC. ELC, earlobe crease; CAD, coronary artery disease.

**Figure 3:**
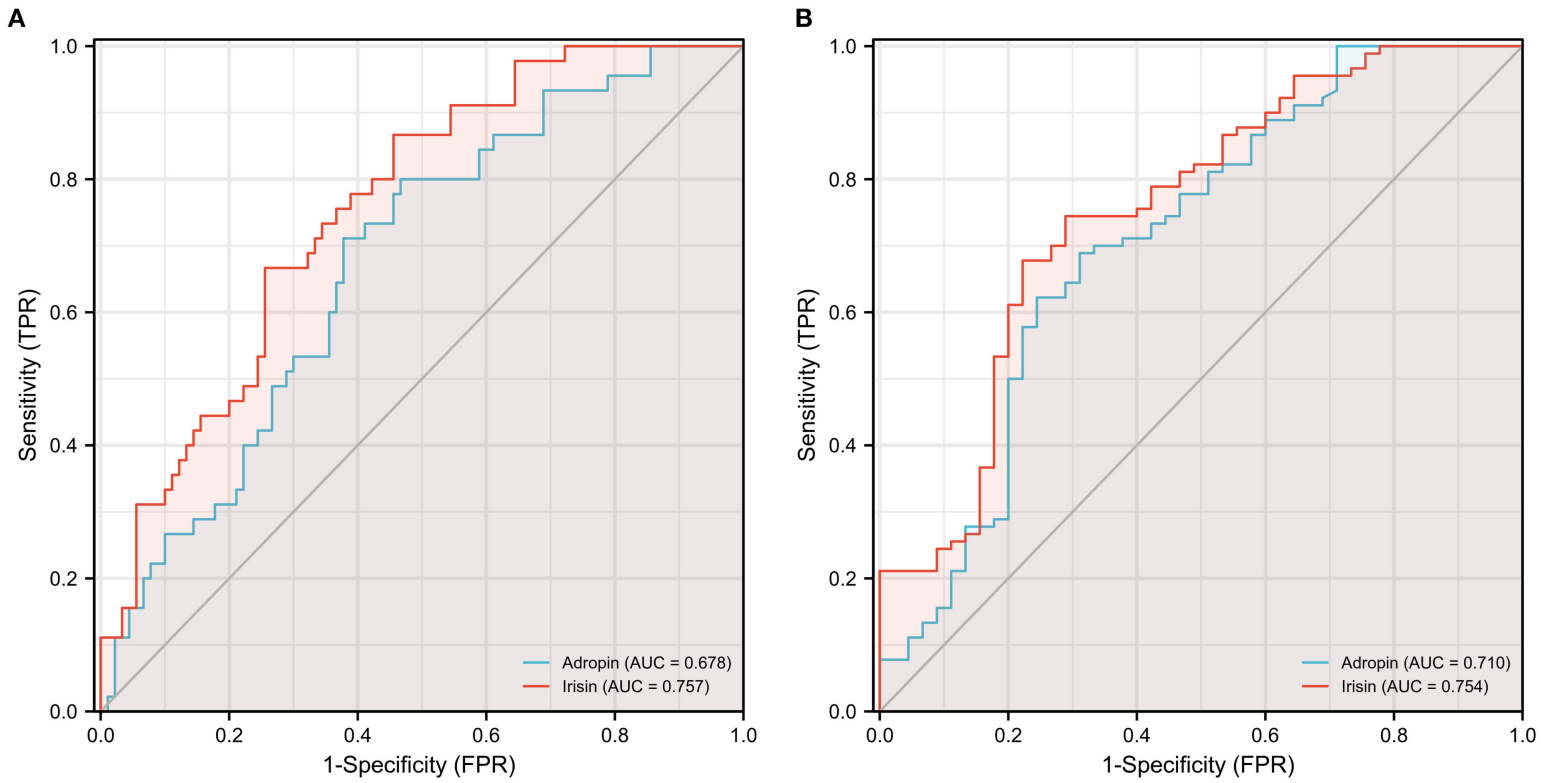
Receiver operating characteristic (ROC) curve analysis discriminating CAD **(A)** and ELC **(B)** patients from healthy control individuals *via* serum adropin and irisin level. ELC, earlobe crease; CAD, coronary artery disease.

To investigate the diagnostic value of adropin in coronary heart disease, we reclassified all participants into CAD and non-CAD groups based on coronary angiographic findings. As shown in Figures 2A,B, both serum adropin and irisin levels of patients in the CAD group were significantly lower than those of the non-CAD group (adropin: 2182.7 ± 132.6 vs. 2945.7 ± 194.5 pg/l, *P* < 0.001; irisin: 175.0 ± 26.1 vs. 222.8 ± 16.4 μg/l, *P* < 0.001). ROC analysis revealed an area under curve score of 0.678 (95% CI: 0.585–0.770) and 0.757 (95% CI: 0.675–0.838) for adropin and irisin levels, respectively. Regarding adropin as an indicator of CAD, a cutoff value of 1778.5 pg/l had 80 and 53% sensitivity and specificity values, respectively. For irisin, a cutoff value of 270.4 μg/l was associated with 86% sensitivity and 54% specificity (Figure 3A). Unfortunately, we did not find correlation between adropin or irisin levels and SYNTEX scores (Supplementary Figure 2).

### Circulating Levels of Adropin and Irisin in Patients With ELC

**Figure 4:**
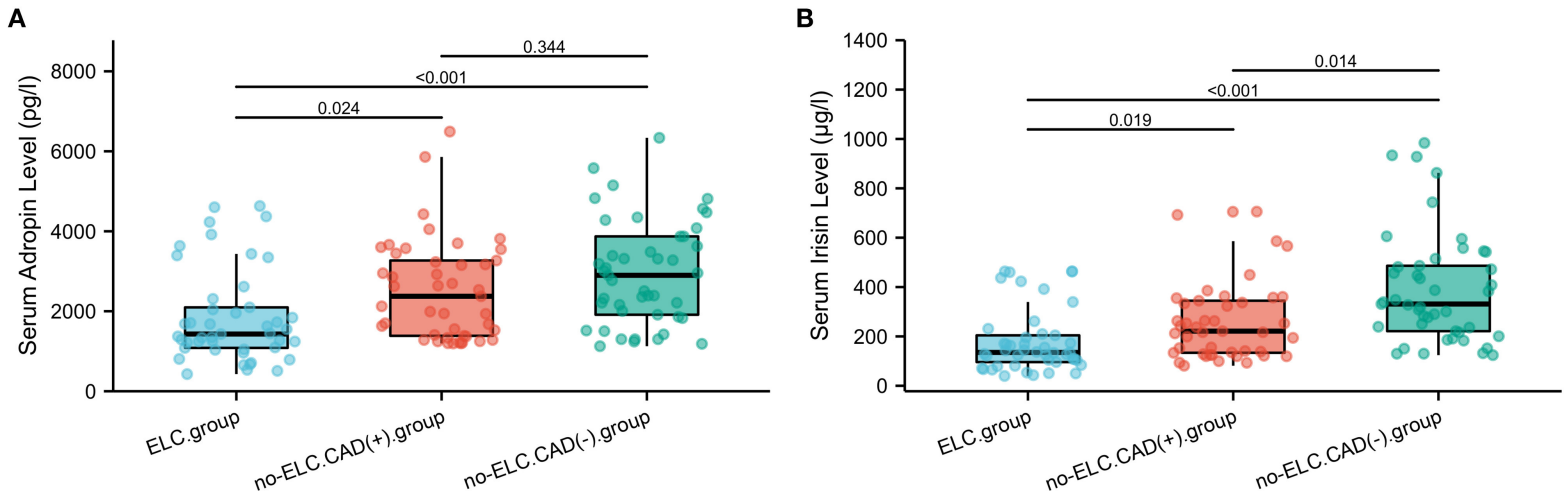
**(A,B)** Comparison of serum adropin and irisin levels between enrolled patients with and without diagonal ELC. ELC, earlobe crease.

As shown in Figure 4A, the mean serum adropin level of the ELC group was 1848.5 ± 176.1 pg/l and of the non-ELC group was 2517.0 ± 187.1 pg/ml. The level in the ELC group was significantly lower than that in the non-ELC group (*P* < 0.001). Interestingly, the serum adropin level of the control group was higher than that of the non-ELC group, but the difference failed to achieve statistical significance (2945.7 ± 194.5 vs. 2517.0 ± 187.1 pg/l, *P* = 0.344). When all participants were categorized into two groups based on the presence of ELC (Figure 2C), serum adropin levels of the ELC group were significantly lower than those of the non-ELC group (ELC group, *n* = 45, 1848.5 ± 176.1 pg/l vs. non-ELC group, *n* = 90, 2731.4 ± 136.1 pg/l, *P* < 0.001). *Via* ROC curve analysis, an adropin cutoff value of 2110.4 pg/l was determined to function as an indicator of ELC with 62.2% sensitivity and 75.6% specificity. The area under the curve for this relationship was 0.71 (95% CI: 0.612–0.809) (Figure 3B).

Meanwhile, the irisin level of the ELC group was significantly lower than that of the non-ELC group, and both ELC and non-ELC group values were lower than that of the control group (179.3 ± 19.2 μg/l vs. 266.3 ± 25.0 μg/l vs. 391.8 ± 33.6 μg/l, *P* < 0.001) (Figure 4B). After reclassification, the irisin level of the ELC group was reduced in accordance with trends for adropin levels (179.3 ± 19.2 μg/l vs. 303.1 ± 18.4 μg/l, *P* < 0.001). ROC curve analysis revealed a cutoff value of irisin of 211.1 μg/l (67.8% sensitivity, 77.8% specificity). The area under the curve was 0.754 (95% CI: 0.664–0.843) (Figure 3B).

## Discussion

The present study revealed that circulating levels of adropin and irisin were significantly reduced in patients with CAD, and the difference between groups was more pronounced in the presence of ELC. Furthermore, both adropin and irisin provided valuable prognostic information for CAD and ELC, indicating that both hormones may contribute to CAD progression.

Several studies have shown that low levels of adropin may be a novel predictor of coronary atherosclerosis. Wu et al. found, in a study of 392 subjects, that serum adropin levels were inversely proportional to Gensini, Friesinger, and SYNTAX scores, and independently associated with coronary atherosclerosis severity (21). A meta-analysis that included 945 participants indicated that serum adropin levels in the CAD group were significantly lower than those in the healthy control group. Differences were observable in a subgroup analysis that included acute myocardial infarction, unstable angina, and stable angina (18). The correlation between irisin and CAD is supported by strong clinical data. A low irisin level has been observed in acute coronary syndrome as well as stable CAD. Low levels of irisin are also inversely related to major adverse cardiovascular event occurrence (19, 22, 23). In accordance with previous studies, our results indicate that irisin level is a valuable biomarker for predicting CAD prognosis, a finding with great potential clinical value.

Several epidemiological and clinical observations offered convincing evidence that the presence of an ELC is a simple sign that may be used to predict atherosclerosis (9, 24); however, the pathophysiological foundation of the association between ELC and CAD remained unknown. A number of hypotheses have been put forth to explain the phenomenon. Shoenfeld et al. (25) demonstrated that biopsy specimens from the earlobes of participants with ELCs revealed some precocious variation among specimens, such as elastin degeneration, atrophic elastic fibers, and a thickened pre-arteriolar wall, features which were also observed in the coronary arteries of patients with CAD. The finding implied that ELC is an external sign of a microangiopathic lesion of terminal vessels. Higuchi et al. (26) showed that telomere length was shortened in tMetS patients with ELC, which suggests excessive telomere loss can be attributed to the formation of ELC. A study by Oda et al. (20), which included 400 consecutive subjects, revealed that the presence of bilateral ELC is often accompanied by endothelial dysfunction, measured by flow-mediated dilation (FMD) and nitroglycerine -induced vasodilation (NID), which suggest that the presence of bilateral ELCs is associated with vascular dysfunction.

In the present study, we show a relatively small but significant association between low adropin or irisin levels and ELC. A plausible interpretation of our results is that adropin and irisin deficiencies prevent the activation of endothelial nitric oxide synthase and promote endothelial dysfunction, which is a common pathological process associated with atherosclerosis and ELC. Interestingly, both hormones have similar prognostic power for CAD and ELC, indicating that endothelial dysfunction due to adropin and irisin deficiencies may be a bridge connecting ELC and CAD.

A strength of this study is that patients aged >70 years and who suffer acute coronary syndrome were excluded, since the presence of the syndrome may interfere with the diagnosis of ELC and affect hormone levels. Thus, confounding due to this issue was minimized in the study. The study does have some limitations worth discussing. First, data collected from normal subjects with ELC were not assessed because we were unable to recruit enough subjects for a meaningful statistical assessment. Moreover, we cannot state a specific causal relationship, and the absence of a suitable animal model has to date severely limited both investigations into the mechanisms. Finally, the sample size of this study was somewhat limited, and the non-invasive vascular function measures such as FMD and NID was lack although it has also been confirmed in several other studies (20). Replication of these results when assessing a larger and more diverse sample and further functional studies to identify the common-cause and cause-effect and are critically important.

In conclusion, findings of this study may enhance our understanding of potential mechanisms that underly ELC and may have some clinical and public health implications.

## Data Availability Statement

The original contributions presented in the study are included in the article/Supplementary Material, further inquiries can be directed to the corresponding authors.

## Ethics Statement

The studies involving human participants were reviewed and approved by Ethics Committee of Shaanxi Provincial People's Hospital. The patients/participants provided their written informed consent to participate in this study.

## Author Contributions

FL and JW designed this research. NW, RZ, ZZ, QW, MM, SM, and FL performed the experiments. QY, RL, and CX analyzed the data. NW and FL drafted the manuscript. All authors contributed to the article and approved the submitted version.

## Funding

This study was supported by the Natural Science Basic Research Plan of Shaanxi Province (No. 2017KJXX-70), Special Financial Grant from China Post-doctoral Science Foundation (No. 2017T100789), Natural Science Foundation of Shaanxi Province (No. 2021SF-329), and National Natural Science Foundation of China (No. 81400328), Project for Sanqin Academic Innovation Team in Shaanxi Province (No. SQ0157).

## Conflict of Interest

The authors declare that the research was conducted in the absence of any commercial or financial relationships that could be construed as a potential conflict of interest.

## Publisher's Note

All claims expressed in this article are solely those of the authors and do not necessarily represent those of their affiliated organizations, or those of the publisher, the editors and the reviewers. Any product that may be evaluated in this article, or claim that may be made by its manufacturer, is not guaranteed or endorsed by the publisher.

## Supplementary Material

Supplementary Figure 1 Comparison of SYNTEX Scores in subgroup analysis on the basis of CAD and ELC.

Supplementary Figure 2 The correlation between serum adropin **(A)** or irisin levels **(B)** and SNYTEX Scores in CAD group.
