# Supplementary figures and images for: Adropin and Irisin Deficiencies Are Associated With Presence of Diagonal Earlobe Crease in CAD Patients
Source: Front Cardiovasc Med. 2021 Oct 12;8:719763. doi: 10.3389/fcvm.2021.719763 (PMC8545887; doi:10.3389/fcvm.2021.719763)

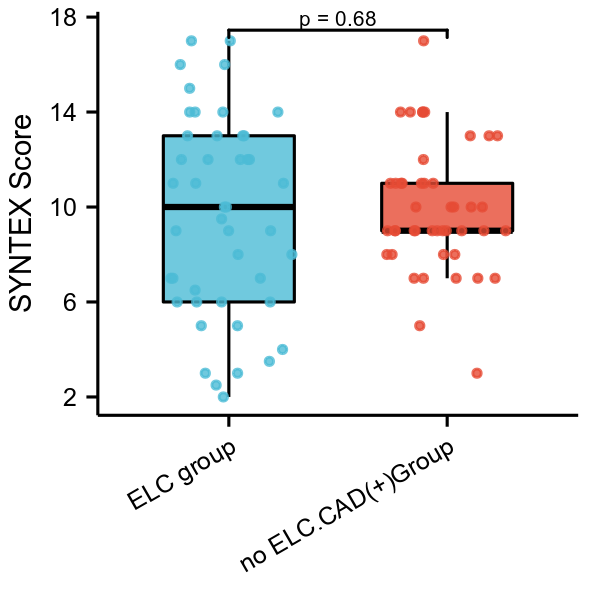

Supplement: Supplementary Figure 1 — Comparison of SYNTEX Scores in subgroup analysis on the basis of CAD and ELC. [file Image_1.TIFF]

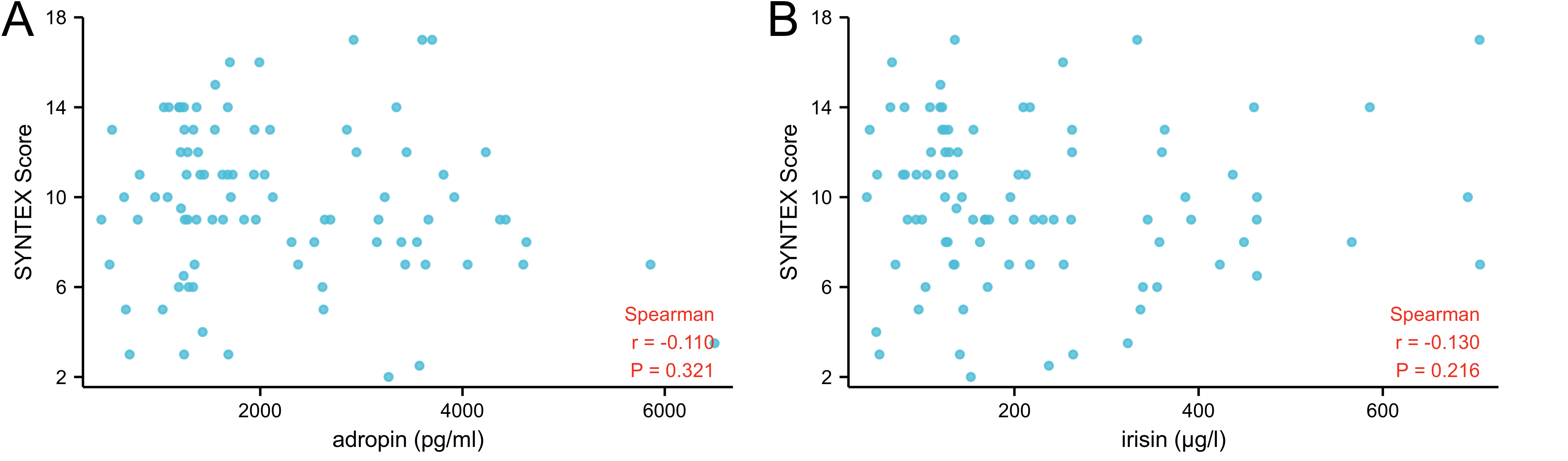

Supplement: Supplementary Figure 2 — The correlation between serum adropin (A) or irisin levels (B) and SNYTEX Scores in CAD group. [file Image_2.TIFF]
